# A long-recommended but seldom-used method of analysis for fall injuries found a unique pattern of risk factors in the youngest-old

**DOI:** 10.1007/s40520-014-0308-x

**Published:** 2015-01-14

**Authors:** Helen Legrand, Mats Pihlsgård, Eva Nordell, Sölve Elmståhl

**Affiliations:** 1 Department of Health Sciences, CRC, Skåne University Hospital, Jan Waldenströms gata 35, 205 02 Malmö, Sweden; 2 Department of Health Sciences, Lund University, Lund, Sweden; 3 Department of Geriatrics, Skåne University Hospital, Malmö, Sweden

**Keywords:** Falls, Injury, Youngest-old, Longitudinal, Community-dwelling, Cox regression for recurrent events

## Abstract

**Background:**

Few studies on fall risk factors use long-recommended methods for analysis of recurrent events. Previous falls are the biggest risk factor for future falls, but few fall studies focus on the youngest-old.

**Aims:**

This study’s objective was to apply Cox regression for recurrent events to identify factors associated with injurious falls in the youngest-old.

**Methods:**

Participants were community-dwelling residents of southern Sweden (*n* = 1,133), aged 59–67 at baseline (median 61.2), from the youngest cohorts of the larger Good Aging in Skåne (GÅS) study. Exposure variable data were collected from baseline study visits and medical records. Injurious falls, defined as emergency, inpatient, or specialist visits associated with ICD-10 fall codes during the follow-up period (2001–2011), were gathered from national and regional registries. Analysis was conducted using time to event Cox Regression for recurrent events.

**Results:**

A majority (77.1 %) of injurious falls caused serious injuries such as fractures and open wounds. Exposure to nervous system medications [hazard ratio (HR) 1.40, 95 % confidence interval (CI) 1.03–1.89], central nervous system disease (HR 1.79, CI 1.18–2.70), and previous injurious fall(s) (HR 2.00, CI 1.50–2.68) were associated with increased hazard of injurious fall.

**Conclusions:**

Regression for recurrent events is feasible with typical falls’ study data. The association of certain exposures with increased hazard of injurious falls begins earlier than previously studied. Different patterns of risk factors by age can provide insight into the progression of frailty. Tailored fall prevention screening and intervention may be of value in populations younger than those traditionally screened.

## Introduction

Falls in older populations are a common issue, with approximately one-third of community-dwelling individuals over the age of 65 falling each year [1–8]. Many of these falls result in injury or death [9–12], and those who fall are more likely to experience worsened function [13, 14] and have a higher risk of transition to sheltered housing [15, 16].

Many modifiable and non-modifiable fall risk factors have been identified in older populations, including environmental, physiological, and pharmacological factors [17, 18]. One factor consistently found to be associated with falls is a history of previous falls, meaning that delaying or preventing early falls may reduce the number of future falls. There is, however, little research focused on falls in the youngest-old, with most studies of fall risk factors focusing on populations with a mean age greater than seventy [17].

Furthermore, to date, most studies on fall risk factors do not use statistical methods that take into account recurrent events, despite longstanding recommendations in the epidemiological literature against simply dividing populations into “fallers” and “non-fallers” [19–21]. Studying total fall burden is of great public health interest, as each fall is associated with costs to the individual and the healthcare system.

The primary objective of this study was to use Cox regression for recurrent events to determine which potential fall risk factors were associated with injurious falls in a relatively young population of individuals.

## Materials and methods

### Design and participants

Using a longitudinal cohort design, we studied the association between injurious falls and selected exposure variables including symptoms, diagnoses, physical performance, and medications in the youngest-old. Participants in the current study were those from the 60- and 66-year-old cohorts (ages 59–61 and 65–67, respectively) from the larger cohort study Good Aging in Skåne (GÅS). GÅS is a longitudinal cohort study of 2,931 individuals 60–93 years of age from five rural and urban municipalities in southern Sweden who were randomly selected on the basis of the National population register. Skåne, which is Sweden’s southernmost county, had 1,136,571 inhabitants in 2001, making up 12.8 % of Sweden’s total population of 8,909,128 inhabitants that year [22].

It was decided for reasons of power, generalizability, and confounding effects to focus solely on the youngest two cohorts in GÅS. These cohorts had higher participation rates and cohort sizes more than twice as large as those of older age groups. Furthermore, focusing on the two youngest cohorts limited birth year effects.

Study visits for the population in this study began January 8, 2001 and ended July 30, 2004. Visits took place at one of the study clinics or at the place of residence if the participant was unable to come to a study clinic. Study size was determined by the size of recruited cohorts in the greater GÅS project. Details of GÅS have been described previously [23].

### Participant flow

During the recruitment period, there were 108,669 residents of Skåne in the target age group of youngest-old. Of the 2,233 initially invited to participate in the study, 1,382 eligible individuals completed the first study visit. The participation rates for men and women were 65.6 and 65.5 %, respectively. The participation rates for the 60- and 66-year-old groups were 62.8 and 68.7 %, respectively. Previous falls were determined in part from entries in a regional database that began in 2001, requiring the removal of 210 individuals with a baseline study visit in 2001 to be able to collect complete data on previous year fall history for the final analysis. Thirty-nine individuals were missing data for one or more covariates and were excluded from the final analysis. This left 1,133 individuals in the final analysis, 83 of whom died during the study follow-up period.

### Measurements

Injurious falls were defined as date of first seeking emergency, inpatient, or specialist care for a medical event associated with an ICD 10 fall-related trauma code (W0-19 or V0-19; with 275 (94 %) W codes and 18 (6 %) V codes). Fall events were sorted by date and ICD code and only the first visit for each unique event was used; patients with more than one unique event had multiple event dates. Data on falls and deaths during the study period were taken from the National Diagnosis Register and Skåne Region’s Healthcare Cost Database. Because outcome status was determined using each individual’s unique identifying number in these registries, loss to follow-up is minimal. The only injurious falls not captured by these databases are those that occurred abroad or those occurring in another region and not resulting in inpatient care.

Exposure variables collected from baseline study examinations and participant surveys were chosen based on their plausible physiological mechanism for increasing fall risk. To limit multiple testing problems, the chosen variables were then grouped into covariates based on similar etiology or potential sequelae, forming 16 covariates composed of 57 sub-variables. Covariate groupings and measurement techniques are specified in Table 1. Baseline exposure to covariates is detailed in Table 2. Two additional covariates (previous falls and sex) were added to the final analysis.

**Table 1:** Covariate composition and data collection methods

Covariate	Sub-variables	Data collection method
Ischemic heart disease	Angina, myocardial infarction, percutaneous coronary intervention, coronary surgery, congestive heart failure	From history (including patient chart) or by physical examination for congestive heart failure
Cerebral hypoperfusion	Arrhythmia, symptoms or signs of orthostatic hypotension	From patient history (including patient chart), self-report of orthostatic hypotension symptoms in the past year, and signs or symptoms during orthostasis testing in clinic
CNS disease	Cerebral infarct, transient ischemic attack, RIND, intracranial hemorrhage, Parkinson disease, epilepsy, multiple sclerosis	From history (including patient chart)
Psychiatric disorder	Dementia, depression, psychosis, sleep disturbances, anxiety	From history (including patient chart)
Sensory impairment	Blindness in one or both eyes, unable to read J4 or larger text on Jaeger chart, subjective vision impairment, tinnitus, hearing impairment on exam, subjective hearing impairment, deafness	From history (including patient chart), patient self-report in questionnaires, and physical exam (including whisper test and Jaeger chart exam with patient’s own visual aids)
Cancer in past 10 years	Inpatient diagnosis code for cancer in the past 10 years	Solely from National diagnosis registry
Obstructive lung disease	Chronic obstructive lung disease, asthma with current treatment	From history (including patient chart)
Musculoskeletal disease	Pain on movement; osteoarthritis of back, hip, knee, or toes; inflammatory arthritis; ankylosing spondylitis; polymyalgia rheumatica	From history (including patient chart)
Dizziness and balance disorders	Subjective feeling of dizziness, subjective tendency to fall, subjective feeling of balance problem, unable to complete 60 s balancing on one leg (right or left) with eyes open, Ménières disease	From patient self-report questionnaires, history (including patient chart), and physical exam (one-leg standing test with patient barefoot, without balance aids, and arms hanging by sides)
Diabetes	Type I or II	From history (including patient chart)
Head trauma	With or without loss of consciousness	from history (including patient chart)
Substance abuse	Self-report	From history
Walking speed below age group median	Maximal or self-selected walking speed below age group median	Walking timed from time subject crosses first marked point on floor, reaches second marked point 15 meters further, turns 180 degrees and walks 15 meters back. Two extra meters provided to start and slow down before and after marked distance. Measured both for self-selected and maximal walking speed. Normal shoes and walking aids allowed
Nervous system medication	Benzodiazepines (ATC classes N03AE, N05BA, N05CD), neuroleptics (ATC class N05A), sedative/hypnotics (ATC class N05C), anticholinergics (ATC class N04A), antidepressants (N06A)	Patient taking any of these medication classes at time of study visit from history (including patient chart)
Antihypertensive medication	ATC class C02, Diuretics (ATC class C03), beta-blockers (ATC class C07), ACE inhibitors and angiotensin receptor blockers (ATC class C09), calcium channel blockers (ATC class C08)	Patient taking any of these medication classes at time of study visit from history (including patient chart)
Opiate medication	ATC class N02A	Patient taking any of these medication classes at time of study visit from history (including patient chart)

**Table 2:** Baseline exposure to covariates in 1,382 youngest-old individuals

Covariate	Exposed *n* (%)	Missing *n* (%)
Ischemic heart disease	159 (13.6)	11 (0.9)
Cerebral hypoperfusion	369 (31.5)	26 (2.2)
CNS disease	58 (4.9)	6 (0.5)
Psychiatric disorder	550 (46.9)	14 (1.2)
Sensory impairment	619 (52.8)	36 (3.1)
Cancer in past 10 years^a^	71 (6.1)	–
Obstructive lung disease	79 (6.7)	12 (1.0)
Musculoskeletal disease	769 (65.6)	6 (0.5)
Dizziness and balance disorders	810 (69.1)	7 (0.6)
Diabetes	88 (7.5)	5 (0.4)
Head trauma	209 (17.8)	14 (1.2)
Substance abuse	62 (5.3)	6 (0.5)
Walking speed below age group median; self-selected or maximum		23 (2.0)
At either self-selected or maximum	264 (22.5)	
At both self-selected and maximum	426 (36.3)	
Nervous system medication^b^	170 (14.5)	–
Antihypertensive medication^b^	326 (27.8)	–
Opiate medication^b^	62 (5.3)	–

^a^Variables from registry that is in theory complete for all participants

^b^Data from medical records and patient interviews that are in theory complete for all participants

### Statistical methods

We conducted a preliminary analysis (*n* individuals = 1,382) using *χ* ^2^ tests with a dichotomous fall outcome to determine which of the sixteen potentially influential covariates to include in the final Cox Regression. This was done to reduce multiple testing problems. The preliminary analysis was carried out using IBM SPSS Statistics for Windows Version 20 (IBM Corp, Armonk, NY, USA).

The final analysis calculated hazard ratios using time to event Cox regression (Andersen–Gill model) [24–26] with participant age as the time scale. Note that using age as the time scale, we view the data as left- as well as right-censored. Formal tests were based on the robust variance estimators defined in Lin and Wei [27]. The six covariates that were found to be statistically significant in the preliminary analysis were included in the final analysis. Sex was added as a covariate. Potential interrelatedness of fall events was taken into account by adding a dichotomous variable for previous falls (during or in the year prior to study participation), for a total of eight variables.

All falls from the year prior to study entry until Dec 31, 2011 were included in the regression; falls in the year prior to an individual’s study start date were only taken into account for the previous falls’ variable. All individuals contributed time at risk from their study entry date until Dec 31, 2011, or until date of death. This analysis was conducted using SAS software version 9.2 (SAS Institute Inc., Cary, NC, USA).

### Statement of human rights

Consent was obtained to use linked data from hospital registries. The study was approved by the regional ethics committee at Lund University and was therefore performed in accordance with the ethical standards laid down in the 1964 Declaration of Helsinki and its later amendments. All subjects provided written consent to participate.

## Results

### Descriptive data

The participants in the final analysis had a median age of 61.2 (mean 63.2) at first study date (range 59.3–68.0); 562 (49.6 %) were male and 571 (50.4 %) were female. Nearly all were community-dwelling (98.1 %). These participants were followed for a total of 9,316.50 person-years (median 8.2, range 0.3–10.0 years). A total of 293 injurious falls occurred among 230 individuals during study follow-up time. The crude event rate was 31.4 per thousand person-years. Of the fall events, 48.5 % caused fractures, 13.7 % caused dislocations, distortions, or injuries of ligaments and tendons, 12.3 % caused an open wound, and 4.8 % caused intracranial bleeding or concussion. In addition to these serious injuries, 22.2 % were associated with contusions and 8.5 % with another type of injury. Some events were associated with more than one injury diagnosis code, yielding a total greater than 100 percent. Only 6 (2.0 %) events were not associated with an injury diagnosis code. Of the fractures, 19.5 % involved the distal forearm or wrist, 14.8 % the humerus, 10.1 % the hip or femur, 8.1 % the foot or ankle, and 47.7 % were in another location. Six events (2.0 %) were associated with more than one fracture.

### Main results

Cox proportional hazards regression of the final covariates yielded statistically significant hazard ratios for the covariates of nervous system medications, central nervous system disease, and previous injurious fall. The results of this analysis are detailed in Table 3.

**Table 3:** Time to event Cox regression for injurious falls based on fall risk covariates in 1,133 youngest-old individuals

Covariate	*p* value	Hazard ratio	(95 % CI)
Male sex	0.05	0.78	(0.61–1.00)
Nervous system medication	0.03	1.40	(1.03–1.89)
CNS disease	0.006	1.79	(1.18–2.70)
Psychiatric disorder	0.23	1.17	(0.91–1.51)
Musculoskeletal disease	0.20	1.19	(0.91–1.55)
Dizziness and balance disorders	0.19	1.21	(0.91–1.61)
Walking speed below age group median; self-selected or maximum			
At either self-selected or maximum	0.92	1.02	(0.74–1.40)
At both self-selected or maximum	0.41	1.13	(0.85–1.50)
Previous fall	<0.001	2.00	(1.50–2.68)

## Discussion

We found that taking nervous system medications, the presence of central nervous system disease, and occurrence of previous injurious fall were associated with increased hazard of injurious fall in a relatively young population. A major strength of this study is the use of statistical analysis for recurrent events, allowing us to include all injurious falls in the analysis rather than only first falls. Few studies of fall risk factors have used this or similar statistical approaches, despite the importance from a public health perspective of analyzing total falls [19, 20].

This method of analysis was straightforward to use with data typically collected during fall studies and the help of widely available statistical software. Using age as time scale in the time to event Cox regression allowed us to take into account age effects over the relatively long follow-up period. Using health records rather than participant recall to determine the status of the outcome variable also eliminated the documented issue of recall inaccuracy [28–30], and allowed us to focus solely on injurious falls.

Individuals experiencing fall injuries were effectively included more than once in the modified Cox regression, albeit with age and previous fall injury status accounted for at the time of each fall. This means that risk profiles for these individuals were more heavily weighted in the analysis, a desired effect since these were associated with a greater number of injurious falls. This is in keeping with the public health goal of studying the overall burden of fall injury.

Although healthcare usage can differ between countries, the pattern of injury severity resulting in seeking medical attention in this group indicates that the outcome is generalizable, with 226 of 293 events (77.1 %) associated with serious injury. A further 61 events (20.8 %) were associated with some other form of injury. The pattern of injuries found in this study is similar to that described in other studies of injurious falls [11, 12] and the pattern of fracture injuries is also consistent with that described in slightly older populations [31, 32].

A limitation of this study is the relatively low event rate in this population compared to older populations [33], which limits the power of our analysis. Additionally, a trade-off of grouping sub-variables to avoid multiple testing issues is the potential for a decreased ability to discover associations of injurious falls with individual sub-variables. Further large studies are needed to corroborate the associations we have found and potentially discover further associations. Our primary outcome focused only on falls resulting in emergency, inpatient, or specialist care. Further studies could include non-injurious falls, since these may also be associated with future injury-causing falls.

Of the covariates considered, some have been associated with injurious falls in older populations but were not found to be associated with injurious falls in our population; for example, balance impairment, female sex, and respiratory disorders [7, 34]. This lack of association in our population despite the large proportions exposed to some of these covariates indicates that younger individuals may not be as susceptible to injuries associated with these risk factors. The concept of frailty, defined as increased “vulnerability to adverse outcomes” [35], is a possible explanation for this finding, which is in keeping with previous studies that have found that older individuals are more prone to injury after falling than younger individuals [9, 36].

## Conclusion

The results of the present study suggest that in the youngest elderly, taking nervous system medications, having central nervous system disease such as stroke or Parkinson disease, and having experienced injurious falls previously are associated with increased hazard of experiencing a subsequent injurious fall. These risk factors have previously been found to be associated with falls in general in older populations [17, 18, 37–39]. This study suggests that the association of these factors with injurious falls may begin earlier than previously studied. Some risk factors previously identified in older populations were not associated with injurious falls in this study, a finding potentially explained by processes of frailty. This study also demonstrates the feasibility of using Cox regression for recurrent events with typical falls’ study data and widely available statistical software. Future studies may consider the benefit of fall prevention screening and intervention in this younger population.
